# Polyhydroxybutyrate (PHB) production from halophiles: a comprehensive review

**DOI:** 10.1186/s40643-026-01119-z

**Published:** 2026-09-01

**Authors:** Nadana Raja Vadivu Ganapathy, Sakshi Singh, Shivansh Ranawat, Navya Hada, Mugesh Sankaranarayanan, Punniyakotti Parthipan

**Affiliations:** 1 Department of Biotechnology and Chemical Engineering, School of Engineering, Faculty of Science, Technology and Architecture, Manipal University Jaipur, Dehmi Kalan, Jaipur, 303007 Rajasthan India; 2 Department of Biotechnology, Vel Tech Rangarajan Dr. Sagunthala R&D Institute of Science and Technology, Chennai, 600062 Tamil Nadu India; 3 Department of Biotechnology, Faculty of Science and Humanities, SRM Institute of Science and Technology, Kattankulathur, Chengalpattu, Chennai, 603203 Tamil Nadu India

**Keywords:** Circular economy, Waste reduction, Sustainability, Environmental conservation

## Abstract

The growing environmental impact of petroleum-based plastics has intensified the search for sustainable and biodegradable alternatives. Polyhydroxybutyrate (PHB), a microbial polyester from the polyhydroxyalkanoate (PHA) family, has emerged as a promising biopolymer due to its biodegradability, biocompatibility, and thermoplastic qualities that rival those of conventional polymers. Halophilic bacteria have attracted considerable attention among PHB-producing microorganisms because they thrive in hypersaline environments, enabling non-sterile cultivation, reducing contamination risks, and facilitating cost-effective downstream recovery by osmotic cell lysis. This review provides a comprehensive overview of recent improvements in PHB production by halophilic bacteria, covering physiological adaptations, metabolic pathways, substrate usage, fermentation techniques, and bioreactor optimisation. Particular emphasis is placed on the use of agro-industrial residues and waste-derived feedstocks as sustainable carbon sources to reduce production costs and increase circular bioeconomy results. Recent advances in downstream processing, such as green extraction technologies, metabolic engineering, CRISPR-based gene editing, and synthetic biology approaches to increasing PHB productivity, are critically reviewed. Additionally, developments in polymer modification, life cycle assessment, industrial scalability, regulatory frameworks, and potential applications in packaging, agriculture, and biomedical engineering are discussed. Despite significant progress, issues such as process economics, saline wastewater control, polymer brittleness, and large-scale commercialisation remain. Integrating halophilic biotechnology with waste valorisation, green recovery technologies, sophisticated metabolic engineering, and circular biorefinery concepts offers a promising strategy for developing economically and environmentally sustainable PHB production systems.

**Graphical abstract:**

**Figure:**
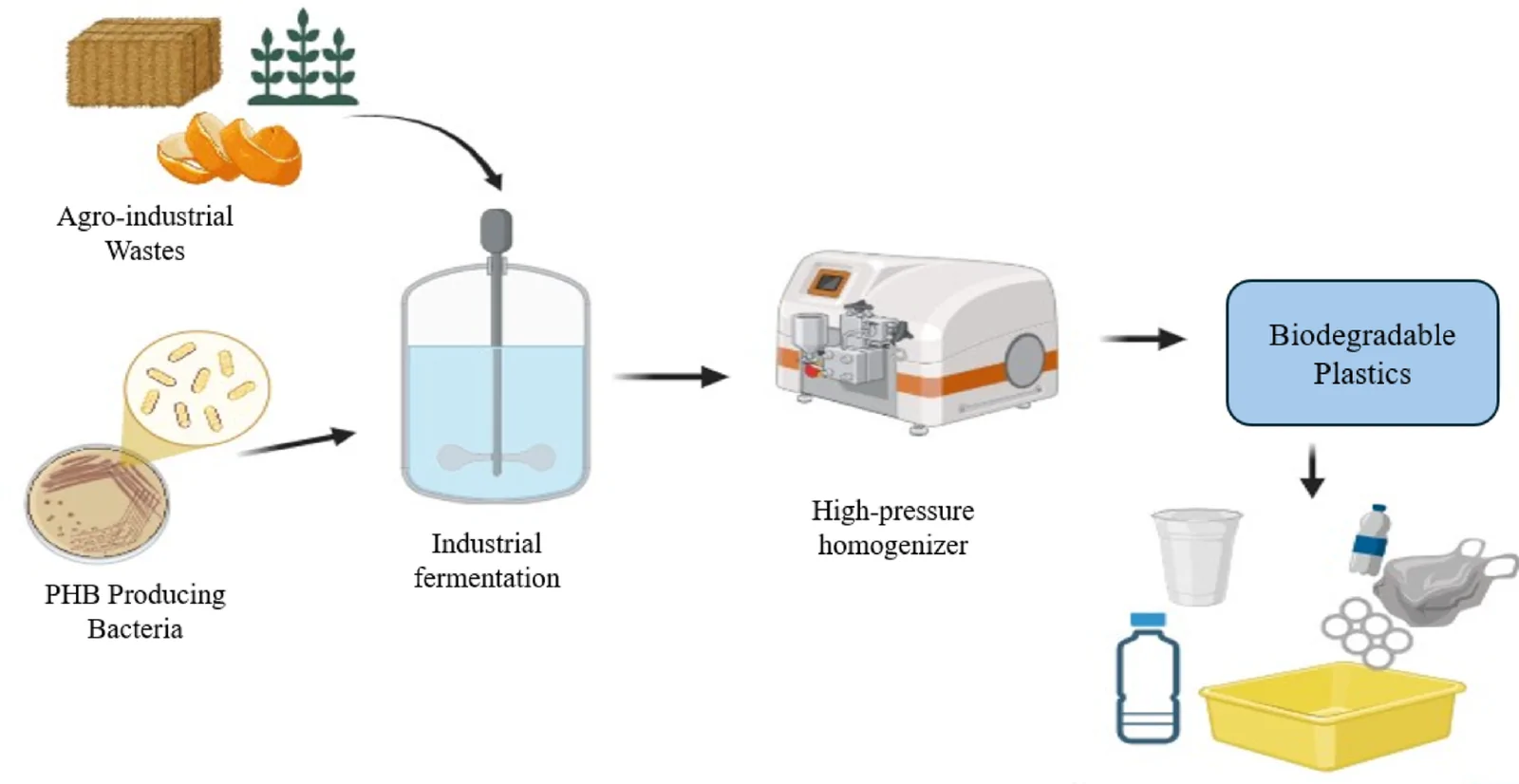

## Introduction

The large and extended use of petroleum-based plastics has had serious environmental implications, including widespread accumulation in terrestrial and marine ecosystems, the production of micro- and nanoplastics, and long-term biological disruptions. According to recent estimates, global plastic manufacturing now exceeds 400 million tons per year, while recycling rates remain below 10%, resulting in persistent environmental contamination (Savoca et al. 2024, Aransiola et al. 2025). Plastic waste has been recorded in protected ecosystems and distant areas, causing major hazards to biodiversity and ecosystem services (Branca et al. 2025). In addition to environmental damage, plastic pollution has been linked to disturbances in the global carbon cycle, exacerbating climate concerns (Yao et al. 2025). These challenges highlight the critical need for sustainable and biodegradable alternatives to conventional plastics.

Biodegradable and bio-based polymers have emerged as a promising solution for addressing plastic pollution and reducing reliance on fossil resources. Polyhydroxybutyrate (PHB), a naturally occurring member of the polyhydroxyalkanoate (PHA) family, has attracted considerable attention because of its complete biodegradability, biocompatibility, and thermoplastic characteristics comparable to those of polypropylene (Li et al. 2025). PHB has high crystallinity (usually 55–80%), as well as good tensile strength and stiffness, making it appropriate for packaging, agriculture, and biomedical applications (Feranc et al. 2025). PHB has been extensively investigated for biomedical applications such as tissue engineering scaffolds and drug delivery systems because of its non-toxic breakdown products and superior biological performance (Mohammadalipour et al. 2025).

Many microorganisms accumulate PHB intracellularly as a carbon and energy storage compound under conditions of excess carbon and limitation of essential nutrients, such as phosphorus or nitrogen. Through strain selection, metabolic engineering, and polymer modification techniques, extensive research has been conducted over the past 20 years to improve PHB productivity, polymer quality, and functional performance (Li et al. 2025, Tanweer et al. 2025). Depending on the microbial species and growth circumstances, reported PHB accumulation levels typically fall between 40 and 70% of the cell dry weight (CDW) (Tanweer et al. 2025). To increase the economic viability and sustainability of PHB production, the utilisation of inexpensive and renewable substrates, such as waste-derived carbon sources and agro-industrial wastes, has also been studied (Lin and Ng 2025).

The large-scale commercialisation of PHB is still constrained by a number of issues, despite these advancements: high production costs, energy-intensive downstream processing, and poorer mechanical and thermal qualities than traditional plastics. Significant research has focused on enhancing PHB performance by blending, composite fabrication, and additive manufacturing techniques to address these restrictions (Li et al. 2025, Feranc et al. 2025). The potential application range of PHB-based materials has increased as a result of these techniques, showing improvements in flexibility, thermal stability, and processability. However, a thorough comprehension that incorporates PHB production processes, material optimisation techniques, and end-use applications is still necessary to enable its transfer from lab-scale research to industrial use. This paper provides a comprehensive overview of PHB, including its environmental impact, microbiological production mechanisms, yield enhancement approaches, material modification methods, and new applications. By critically reviewing prior developments and presenting limits, this analysis aims to identify the main barriers and future approaches required to promote PHB as a feasible and sustainable substitute for petroleum-based plastics.

## Characteristics and structure of Polyhydroxybutyrate (PHB)

### Chemical structure and physical characteristics

Poly(3-hydroxybutyrate) (PHB) is a well-studied member of the polyhydroxyalkanoate (PHA) family that is synthesised intracellularly by a variety of microorganisms as a carbon and energy storage compound in nutrient-limited environments with high carbon availability (Majerczak et al. 2022). PHB is a linear aliphatic polyester with repeating units of (R)-3-hydroxybutyric acid connected by ester linkages. The polymer’s unique ester (− COOR) functional groups and methyl side chains (CH₃) significantly impact its physicochemical properties (McAdam et al. 2020). Depending on the producing organism and growth conditions, PHB can account for up to 80% of the cell dry weight and is classed as a short-chain-length PHA (Turco et al. 2021).

PHB has a high degree of crystallinity (55–80%) and a melting temperature of around 175–180 °C, resulting in good mechanical strength and barrier characteristics equivalent to polypropylene (McAdam et al. 2020). In addition, PHB exhibits piezoelectric activity, expanding its potential uses in biomedical devices and flexible electronics (Motiee et al. 2023). Nonetheless, despite these attractive properties, PHB’s industrial application is restricted by its inherent brittleness, narrow thermal processing window, high crystallinity, and relatively slow degradation rate when compared to other biodegradable polymers such as polylactic acid (PLA) and poly(lactic-co-glycolic acid) (PLGA) (Martínez-Herrera et al. 2021). As a result, significant attempts have been made to address these limitations and increase processability through polymer blending, copolymerization, and nanocomposite synthesis.

PHB is entirely biodegradable and undergoes a multistep degradation process that includes depolymerisation into oligomers and monomers, microbial assimilation into biomass, and ultimately mineralisation into carbon dioxide and water under aerobic conditions (Gasparyan et al. 2023). Several factors significantly influence degradation kinetics, including polymer crystallinity, surface roughness, hydrophobicity, molecular weight, and environmental conditions (Meereboer et al. 2020). Recent studies have shown that PLA/PHB blends can be completely degraded under home composting conditions due to the creation of biodegradable copolyester networks (Fogašová et al. 2022). Similarly, under certain environmental conditions, PHB-based composites have shown 100% biodegradation in about 30 days (Manikandan et al. 2020).

From an environmental standpoint, PHB has significant advantages over traditional petroleum-based polymers. Despite its relatively high production cost, PHB has better oxygen and moisture barrier qualities than comparable synthetic polymers, making it more suitable for packaging applications (Markl 2018). Furthermore, life cycle assessments have shown that PHB derived from renewable feedstocks, notably cyanobacterial biomass and agro-industrial waste, can significantly reduce greenhouse gas emissions while improving overall sustainability (Das et al. 2021, Frehner et al.). Importantly, PHB may be chemically valorised using thermolytic distillation, providing crotonic acid with conversion efficiencies of up to 92% and therefore facilitating circular bioeconomy techniques and high-value product recovery (Samorì et al. 2022).

### Industrial and biomedical applications

PHB’s unique mix of biodegradability, biocompatibility, and thermoplastic characteristics has allowed it to be used in a wide range of industrial and biomedical applications. Industrially, PHB has been studied for the production of biodegradable packaging materials, agricultural films, disposable items, and environmentally friendly polymers. Several microorganisms have shown strong PHB synthesis capabilities utilising inexpensive and renewable substrates. For example, *Agromyces indicus* produced 4.2 g/L PHB under optimal conditions (Adnan et al. 2022), but *Azotobacter vinelandii* accumulated 37.7% PHB while also synthesising stable PHB nanoparticles from grape-processing residues (Andler et al. 2024). Bacillus spp. cultured on dairy effluent produced 3.6 ± 0.15 g/L PHB, demonstrating the potential for combining waste valorisation with sustainable biopolymer synthesis (Narwal et al. 2025).

PHB has received a lot of attention in the biomedical field due to its outstanding biocompatibility, bioresorbability, and nontoxic breakdown products. PHB-based materials have been studied for application in surgical sutures, fixation devices, wound dressings, skin grafts, and controlled drug delivery systems (Raza et al. 2020). Furthermore, porous PHB scaffolds have shown great promise in tissue engineering applications such as vascular, neural, cardiac, and bone tissue regeneration, thanks to their advantageous mechanical characteristics and porous design (Kalia et al. 2023). PHB and similar PHAs have also been studied as medical implants, tissue adhesives, and biodegradable medical disposables (Ansari et al. 2021). D-3-hydroxybutyric acid, PHB’s natural breakdown product, is physiologically compatible, making it more suitable for long-term biomedical applications, particularly controlled medication release and bone tissue engineering (Mohan et al. 2021, Pryadko et al. 2022). Considering these promising uses, the general commercialisation of PHB-based products remains hampered by high production costs, brittleness, and processing constraints. To fully realise PHB’s promise in sustainable and biomedical applications, future research should focus on cost-effective manufacturing processes, material modification, and large-scale industrial implementation.

## Halophiles as PHB producers and their metabolic pathways

### Halophiles and their adaptation mechanisms

As shown in Table 1, halophiles are extremophilic bacteria that flourish in high-salt environments due to physiological and molecular adaptations. By building up osmotic solutes to counteract external osmolarity, they stop cytoplasmic dehydration (Linekha et al. 2024). These bacteria are categorised as mild (2–5%), moderate (5–20%), or extreme halophiles (20–30%) based on their tolerance to NaCl (Oren and Salinibacter 2013). The genera *Haloferax*,* Halomonas*, and *Halobacterium* are representative. The genomes of *Halarchaeum* members range in size from 2.6 to 3.1 Mbp, and *Hla. Solikamskense* DSM 26612T and *Halarchaeum nitratireducens* JCM 16331T are highly comparable (Wang et al. 2024). Over 1000 halophilic species were found in a global survey, with 21.9% being archaea, 27.9% being eukaryotes, and 50.1% being bacteria (Loukas et al. 2018). Owing to carotenoids that offer photoprotection, the majority are pigmented and gram-negative; 77 of the 1351 isolates from the Arabian coast of India had pigmentation (66.23% yellow, 20.78% orange, and 12.99% red) (Moopantakath et al. 2021).

**Table 1:** PHB production by moderate and extreme halophilic microorganisms under different cultivation conditions

Organism name	Halophilic type (moderate/extreme)	Optimal NaCl concentration (%)	PHB yield (g/L)	Cultivation conditions (pH, temperature)	Carbon source used	References
*Halomonas boliviensis*	Moderate	10%	4.89 g/L	7.5 pH, 35 °C	Glucose	(Arcila-Echavarría et al. 2025)
*Halomonas boliviensis* *LC1*	Moderate	2.5%	5.04 g/L	7.5 pH, 35 °C	Starch hydrolysate	(Rivera-Terceros et al. 2015)
*Halomonas cerina / Halomonas sp. YK44*	Moderate	4	8.98 g/L	7.0 pH, 25 °C	Galactose	(Jung et al. 2022)
*Halomonas sp. TD01*	Moderate	6	24.0 g/L	7.0 pH, 35 °C	Glucose	(Tan et al. 2011)
*Halomonas elongata*	Moderate	10	21.2 g/L	7.0 pH, 35 °C	Glucose	(Leandro et al. 2023)
*Haloferax mediterranei*	Extreme	10	20.5 g/L	7.0 pH, 37 °C	Sesame seed wastewater + glucose	(Alsafadi et al. 2023)
*Chromohalobacter canadensis*	Moderate	10%	2.1 g/L	7.3 pH, 36 °C	Fructose	(Radchenkova et al. 2020)

PHB yields are reported as either g/L or percentage of cell dry weight (CDW) according to the original literature. Table 1 shows that *Halomonas* species have higher PHB productivity at moderate salinity (5–10% NaCl), making them appropriate for industrial-scale production. Their ability to use a variety of carbon sources and grow in non-sterile settings leads to increased production and economic viability. Extreme halophiles, such as *Haloarcula* and *Halorubrum*, may survive high salinity but have reduced biomass productivity and require more severe cultivation conditions.

Athalassohaline (diverse ionic composition) and thalassohaline (NaCl-dominated, e.g., salt lakes) are two types of halophilic ecosystems (Shukla et al. 2020). Particular dinucleotide abundance profiles are one example of genomic adaptations (Paul et al. 2008). Osmosensing in *Pontixanthobacter* and *Allopontixanthobacter* is controlled by BCCT family transporters (Liu et al. 2019). At 20% NaCl, strains SL3, SL32, SL35, J8W, and PU62 promoted plant development, whereas strains GREB3, GRRB3, and SPSB2 improved plant salt resistance through stress gene modification (Aizaz et al. 2023).

Without interfering with metabolism, halophiles collect compatible solutes such as proline and glycine betaine (Willey et al. 2020). Two-component systems are used in their adaptation, in which response regulators control gene expression and sensor kinases identify osmotic stress (Salma et al. 2020). While *Pseudoalteromonas phenolica* increases proline (13.3-fold) and betaine under stress (Song et al. 2020), *E. halophiles* EGI 80432T switches from a salt-in approach (4% NaCl) to compatible solute production (9% NaCl) (Chen et al. 2021). In saline agriculture, these halotolerant plant growth-promoting bacteria (PGPB) function as biofertilizers (Sagar et al. 2022).

### Industrial advantages of halophiles in PHB production

Halophiles offer significant benefits for the generation of PHB. Open and nonsterile fermentation is made possible by high salinity, which inhibits contamination (Liu et al. 2019, Stanley et al. 2021, Obruča et al. 2022), decreasing the need for specialist fermenters and the expense of sterilisation (Obruča et al. 2022). Their resilience enhances the economic viability of large-scale biopolymer production by supporting open and continuous systems using mineral-rich seawater (0.8–2 g/L Mg, Ca, and K) (Montemurro et al. 2022).

### PHB biosynthesis and regulation in halophiles

**Fig. 1:**
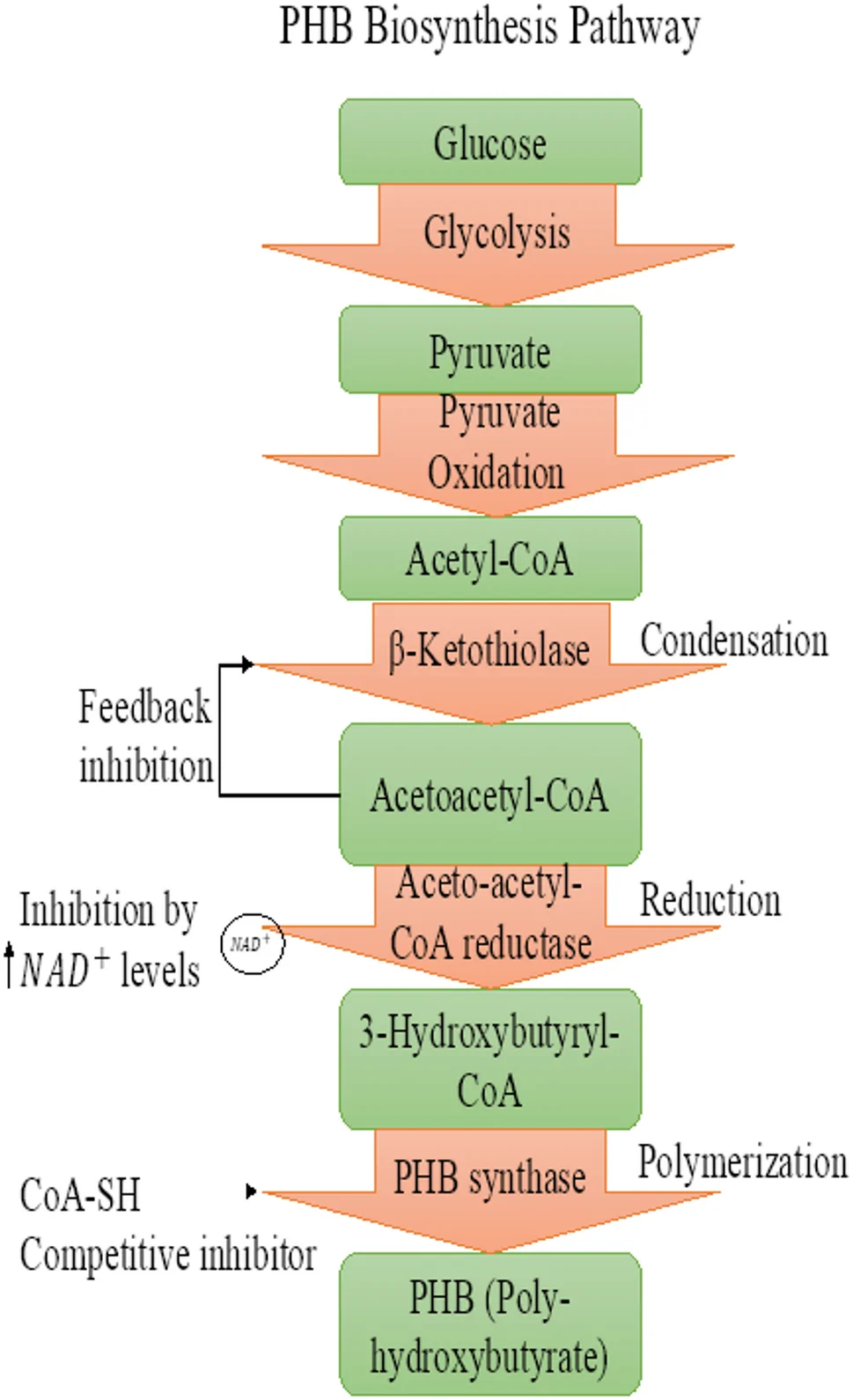
Metabolic and enzymatic pathways of PHB biosynthesis from glucose via acetyl-CoA

Phasins (PhaP), PHA synthase (PhaC), and depolymerases (PhaZ) are among the proteins that surround the insoluble, spherical granules of PHB, which are produced in the cytoplasm (Liu et al. 2020, Wang et al. 2025). Pyruvate is produced via the Entner–Doudoroff pathway or glycolysis, which catabolizes glucose (Kong et al. 2024). PHB synthesis is driven by three enzymes, with conserved cysteine and histidine residues necessary for catalysis (Bhaskar et al. 2020, Müller-Santos et al. 2021). β-ketothiolase (PhaA) condenses two acetyl-CoA molecules into acetoacetyl-CoA (Liu et al. 2020, Ma et al. 2020, Meng et al. 2025). Acetoacetyl-CoA reductase (PhaB) can be improved through genetic engineering (Singh et al. 2020). It uses either NADH or NADPH to convert acetoacetyl-CoA to (R)-3-hydroxybutyryl-CoA, preferring NADH (Olavarria et al. 2021). CoA is released when (R)-3-hydroxybutyryl-CoA is polymerised into PHB by PHA synthase (PhaC) (Sivashankari et al. 2023). Substrate-to-CoA ratios determine its activity (Velázquez-Sánchez et al. 2020, Carmen Fuentes et al. 2024). The phaCAB operon and related genes (phaP, phaZ, phaY, phaR, and phaD) control PHB metabolism. While PhaR suppresses PhaP until PHB accumulation occurs, PhaP stabilises the granules (Velázquez-Sánchez et al. 2020, Esposito et al. 2023, Mitra et al. 2022). Recycling is mediated by the depolymerases PhaZ and PhaY (Liu et al. 2020, Kurabi et al. 2022). When there is enough carbon available, PhaD promotes transcription (Eugenio et al. 2010), and when there is a nitrogen shortage, the sigma factor RpoN controls metabolism (Liu et al. 2020). As shown in Fig. 1, these metabolic and genetic processes work together to guarantee dynamic PHB synthesis in halophiles in response to nutritional status and environmental stress.

## Utilising substrates, optimising bioprocesses, and fermentation techniques

Halophilic bacteria can produce polyhydroxybutyrate (PHB) from a variety of carbon substrates because of their exceptional metabolic flexibility. Halophilic PHB production has been made more economically and environmentally feasible by the effective use of both traditional substrates (glucose, acetate, and glycerol) and inexpensive industrial or agro-waste streams (molasses, whey, orange peel, and algal biodiesel waste residue).

### Substrate utilization

In a few of the halophilic strains, glucose continues to be the predominant carbon source, facilitating substantial PHB accumulation. For example, under 8–10% NaCl, *Halomonas shrimpha* IBTH01 and *Marinobacter haeunpha* IBTM02 produced 4.47 and 4.25 g/L PHB, respectively, whereas *Halomonas bluephagenesis* and *H. rowanensis* produced up to 82% DCW as PHA (Kong et al. 2024, Faulkner et al. 2023). With a polymer productivity of 0.39 g L⁻¹ h⁻¹, *Halomonas elongata* 1H9T achieved 21.2 g/L P(3HB) (Leandro et al. 2023). Additionally, glycerol is a useful substrate that allows PHB accumulation in *Halomonas sp*. KM-1 to reach 63.6% DCW (Hammami et al. 2022). Vibrio alginolyticus produces 73.7% PHA yield with 4.69 g/L biomass, whereas *Haloferax mediterranei* effectively uses volatile fatty acids, resulting in a PHBV of 2 g/L (Parroquin-Gonzalez and Winterburn 2023, Balan et al. 2025).

Agro-industrial waste has been effectively turned into cash. PHB accumulation of up to 72% DCW was made possible by lignocellulosic hydrolysates from wheat straw (Vlaeminck et al. 2022). Without further nutrients, *Halomonas ventosae* and *H. daqingensis* are sustained by algal biodiesel waste residue (Dubey and Mishra 2021). Similar PHB yields to those of glucose media were obtained from vegetable and citrus wastes, sugarcane molasses, and salted whey; at high biomass, *Haloferax mediterranei* achieved 48.6% PHBV (Longo et al. 2024, Abdelrahman et al. 2024). *Bacillus cereus* LB7 and *Burkholderia gladioli* 2S4R1 were able to achieve productivities of 0.309–0.387 g L⁻¹ h⁻¹ in paddy straw hydrolysates (Naitam et al. 2022). As shown in Table 2, these investigations collectively demonstrate the ability of halophilic microbes to generate PHB from a variety of inexpensive, sustainable, and varied feedstocks.

**Table 2:** Agro-industrial and waste-derived substrates for PHB production by halophiles

Halophilic species	Substrate used	Type of substrate	PHB yield reported	Cost-effectiveness	Scalability	Reference
*Halomonas* sp. *YLGW01*	Mixed sugars	Low-cost sugars	83 wt% PHB of CDW	High	High	(Park et al. 2021)
*Salinivibrio* sp. *M318*	Waste fish oil, glycerol	Industrial lipid waste	56 wt% PHB of CDW	High	Moderate–High	(Thuoc et al. 2019)
*Halomonas* sp. *SF2003*	Glucose	Refined sugar	61 wt% PHB of CDW	Moderate	High	(Thomas et al. 2020)
*Salinivibrio* spp.	Palm oil mill effluent	Agro-industrial effluent	48 wt% PHB of CDW	High	Moderate	(Rizki et al. 2023)
*Halomonas* spp. *YLGW01*	Fructose	Refined sugar	90 wt% PHB of CDW	Moderate	High	(Park et al. 2020)
*Halomonas boliviensis LC1*	Quinoa stalk hydrolyzate	Agricultural residue	54 wt% PHB of CDW	High	Moderate–High	(Miranda et al. 2023)
Recombinant *Halomonas elongata* A1	Wheat straw hydrolysate	Lignocellulosic waste	73 wt% PHB of CDW	High	High	(Liu et al. 2021)
Halophilic bacteria	Waste cooking oil/fish oil	Lipid waste	60–72 wt% PHB	High	Moderate–High	(Loan et al. 2022)
*Halomonas bluephagenesis*	Glucose (high nitrogen medium)	Refined sugar	75 wt% PHB of CDW	Moderate	Very high	(Zhang et al. 2024)

The cost-effectiveness and scalability given in Table 2 were assessed using numerous parameters mentioned in the literature, such as substrate availability, pretreatment needs, PHB productivity, process complexity, and confirmation of a pilot-scale or industrial application. Waste-derived substrates, such as lignocellulosic hydrolysates, waste oils, and agro-industrial wastes, were widely regarded as extremely cost-effective due to their low cost and contribution to waste valorization. Similarly, procedures that exhibit stable large-scale or pilot-scale production were classified as having good scalability. Waste-derived substrates, such as lignocellulosic hydrolysates, waste oils, and agro-industrial residues, drastically lower production costs while promoting circular bioeconomy strategies. Variations in substrate composition, as well as the demand for pretreatment, may have an impact on microbial growth and PHB buildup.

### Fermentation strategies

Table 3 lists the batch, fed-batch, and continuous fermentation methods that have been used to demonstrate halophilic PHB synthesis. While fed-batch techniques enable regulated substrate feeding, larger cell densities, and improved PHB accumulation, batch approaches are appropriate for strain screening and initial tuning. In nonsterile, high-salinity environments, continuous systems offer reliable operation, improved volumetric productivity, and constant polymer quality (McAdam et al. 2020).

Nutrient limitation, pH, and salinity all significantly affect PHB synthesis. For example, *Haloferax mediterranei* grows best at approximately 20% (w/v) salt and pH 7.25, but it can tolerate 10–35% salt and pH 6.25–8.25 (Matarredona et al. 2021). Many halophiles have wide tolerance ranges. PHB accumulation is frequently maximized by moderate salinity (4–10%) and slightly alkaline pH (~ 8) (Carlozzi et al. 2020). As observed in *Bacillus megaterium uyuni* S29, where phosphorus limitation with KCl supplementation increased P(3HB) productivity to 0.25 g L⁻¹ h⁻¹, nutrient constraints, especially nitrogen or phosphorus, further increase polymer productivity (Schmid et al. 2021).

**Table 3:** Fermentation strategies for increased PHB production in halophiles

Mode (batch/fed-batch/continuous)	Bacterial halophilic strain used	Key parameters (salinity, pH, and temp)	Productivity	Advantages/challenges	Reference
Fed-batch (pilot scale)	*Halomonas bluephagenesis*	High salinity (~ 60 g/L NaCl); pH ~ 7.0; ~37 °C	High pilot-scale PHA productivity	Nonsterile cultivation: oxygen transfer optimization needed	(Ye et al. 2018)
Batch	*Halomonas elongata* (local strain)	High salinity, neutral pH; mesophilic	Moderate PHB accumulation	Robust growth: lower productivity than engineered strains	(Wen et al. 2024)
Batch/Fed-batch	*Halomonas* spp.	High salinity; pH ~ 7; ~37 °C	Improved PHB productivity with enhanced oxygen	Higher yield; increased aeration cost	(Ouyang et al. 2018)
Continuous (open)	Recombinant *Halomonas campaniensis* LS21	Seawater salinity; neutral pH; 30–37 °C	Stable continuous PHA productivity	Open continuous process: genetic stability required	(Yue et al. 2014)
Fed-batch	*Halomonas boliviensis*	Moderate salinity; pH ~ 7; 35 °C	High PHB accumulation (> 50% CDW)	High cell density; fed-batch control complexity	(Quillaguamán et al. 2008)
Batch (nonsterile)	*Halomonas* spp. *WZQ-1*	High salinity, neutral pH; mesophilic	Sustainable PHB production from biowaste	Low contamination risk; feedstock variability	(Wang et al. 2025)
Batch	Halophilic bacterial isolates	High salinity, neutral pH; mesophilic	Variable PHB productivity	Local strain potential, strain variability	(Patience et al. 2021)
Batch	*Salinivibrio* spp. TGB4, TGB19	High salinity, neutral pH, 30–37 °C	Efficient PHB from acetate and butyrate	VFA utilization; substrate inhibition at high VFA	(Tao et al. 2022)
Batch	*Halomonas boliviensis* LC1	Moderate salinity; pH ~ 7; 35 °C	PHB up to ~ 60% CDW	Stable moderate halophile; batch productivity limits	(Quillaguamán et al. 2006)
Batch	Moderate halophilic bacterium	High salinity; neutral pH	PHB synthesized with aqueous extraction	Easier downstream recovery; lower yield	(Chen and Zhang 2014)
Batch	*Halomonas salina*	High salinity, neutral pH, mesophilic	PHB + ectoine coproduction	Dual product strategy; carbon flux competition	(Chen and Zhang 2014)

Fed-batch fermentation often produces more PHB due to controlled food supply and increased cell density. Continuous fermentation improves scalability and production but necessitates meticulous process control and genetic stability. Batch systems remain useful for strain screening and laboratory optimization.

### Bioreactor design and scale-up

PHB productivity and yield are strongly affected by the reactor layout. While airlift reactors (ALRs) and loop modifications enhance gas–liquid mass transfer and lessen shear stress, stirred-tank reactors (CSTRs) are commonly used because of their operating flexibility (Safaeian et al. 2023). *Haloferax mediterranei* grown on VFAs in a CSTR produced stable PHBV (~ 30 mol% 3HV) with productivities of 0.87–1.43 g L⁻¹ h⁻¹ for 100 h, confirming that scale-up investigations can operate continuously (Parroquin-Gonzalez and Winterburn 2023). Similarly, PHB levels of 56–61 weight% were attained by *Halomonas boliviensis* cultivated in ALRs with starch or glucose/xylose hydrolysates, indicating scalability (Rivera-Terceros et al. 2015). Overall, the combination of inexpensive substrates, ideal salinity and pH levels, suitable fermentation techniques, and reactor designs makes halophilic bacteria reliable, profitable, and sustainable platforms for industrial PHB production.

## Downstream recovery and extraction strategies

### Cell harvesting and lysis

**Fig. 2:**
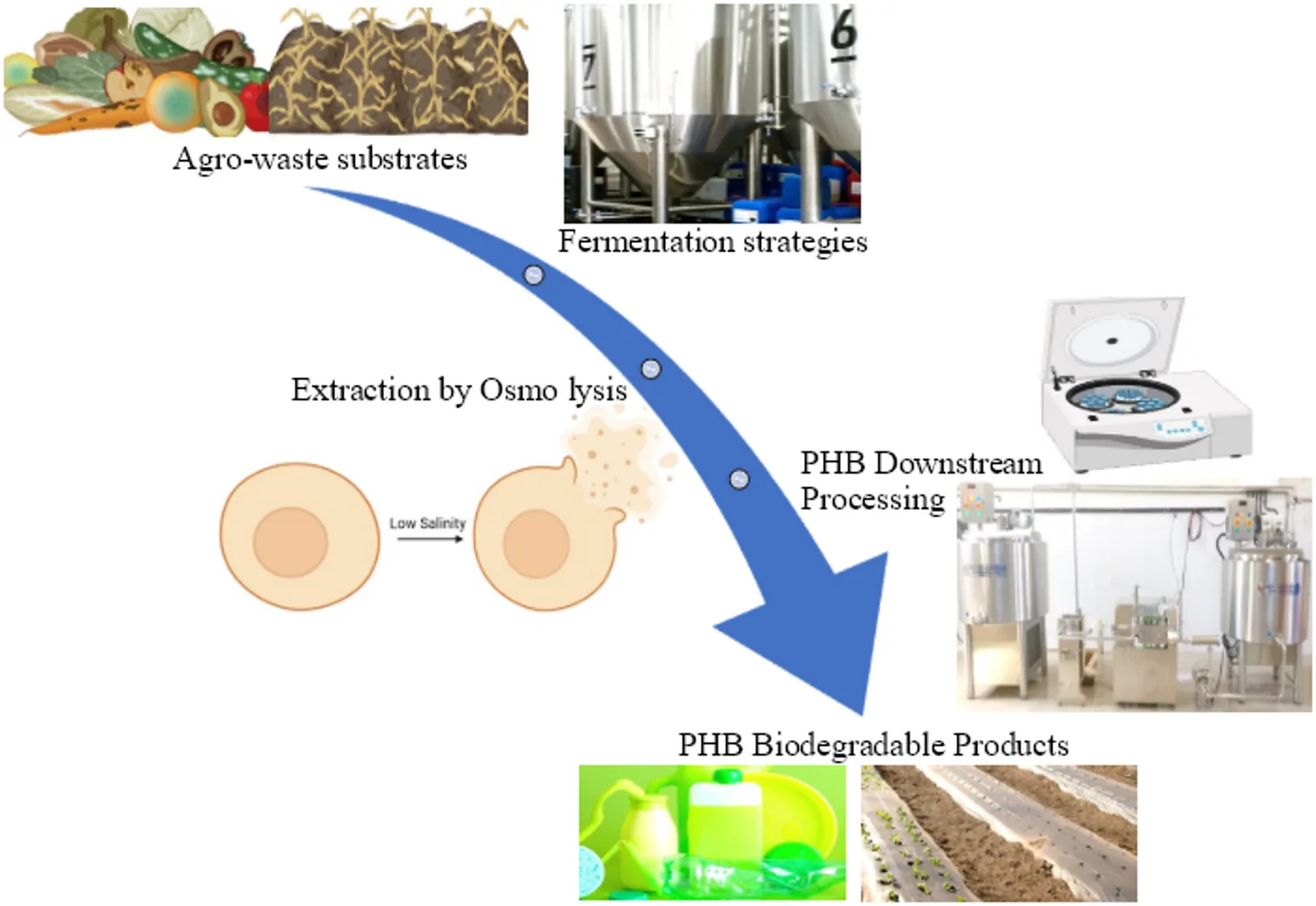
Integrated process flow for PHB production from agro-waste via halophilic fermentation and osmotic extraction

The downstream processing (DSP) of polyhydroxyalkanoates (PHAs), especially polyhydroxybutyrate (PHB), from halophilic microorganisms is complicated by high-salinity fermentation broths. As illustrated in Fig. 2, these broths change the properties of the cell surface and increase the viscosity of the medium, which decreases the centrifugation and filtration efficiency (Novackova et al. 2022). Energy-efficient biomass recovery is made possible by the inherent susceptibility of halophiles to osmotic shock, which can be further improved by the generation of self-flocculating strains (Kalia et al. 2024).

Cell lysis techniques, which are sometimes enhanced with surfactants or mechanical techniques, take advantage of halophiles’ susceptibility to hypotonic environments. SDS is recoverable by KCl precipitation for economic viability, and hypotonic shock enhances membrane breakage (Novackova et al. 2022). While ultrasound-assisted solvent-free extraction enhances polymer crystallinity and thermal stability, microwave-assisted lysis with EDTA results in 93.8% PHB recovery and 97.2% purity (Balakrishna Pillai et al. 2018). Although they are less scalable, mechanical‒chemical techniques such as sonication and bead‒assisted vertexing result in moderate recovery (~ 35–45%) (Bhat et al. 2024). Phage-assisted or planned lysis are examples of emerging techniques that seek to simplify DSP, lower costs, and preserve polymer integrity (Pérez-Rivero et al. 2019).

### Conventional solvent-based extraction

Because of their excellent solubilization efficiency and purity (~ 0.79–0.85 g PHA/g extract), traditional solvent extraction techniques utilizing acetone, dimethyl carbonate (DMC), or chloroform are still often used (Longo et al. 2024, Montiel-Jarillo et al. 2022). However, their industrial sustainability is limited by toxicity, environmental effects, and solvent recyclability. Scalable alternatives are provided by high-boiling, low-toxicity solvents, including 1,2-propylene carbonate and acetone–ethanol combinations, whose viability is supported by process modeling (Abate et al. 2023, Fei et al. 2016).

**Fig. 3:**
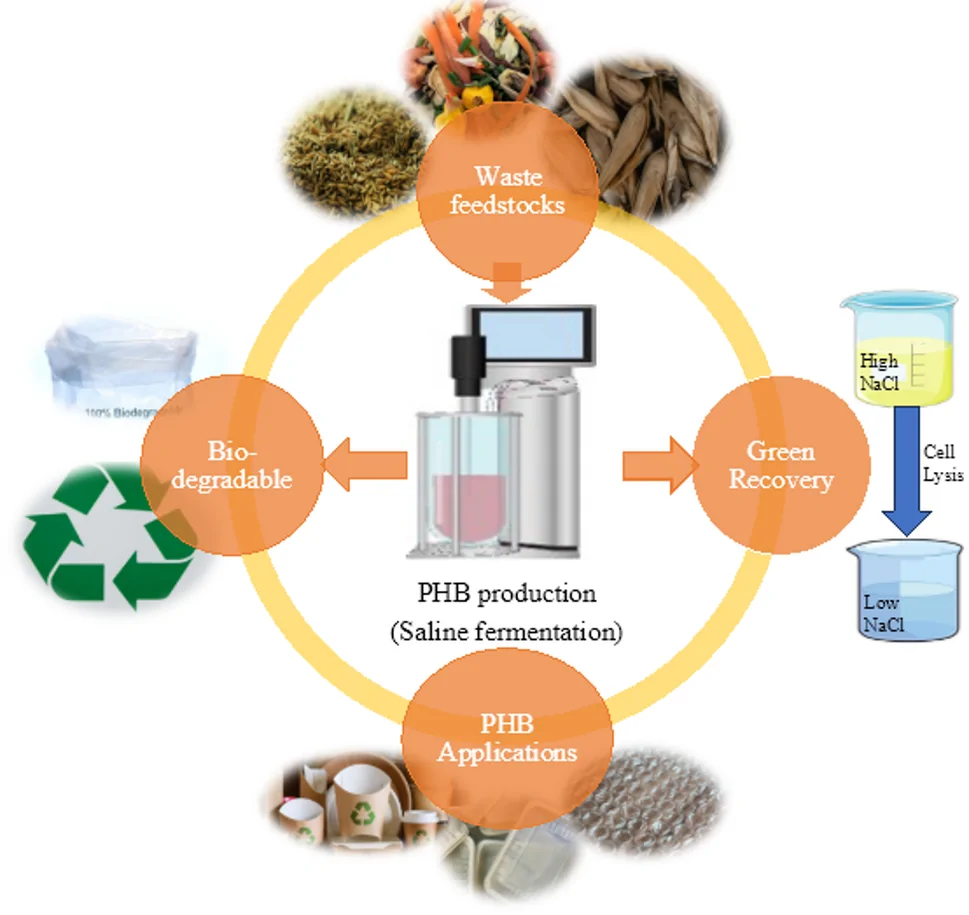
Schematic representation of sustainable PHB production via halophilic saline fermentation and green recovery

Green recovery strategies, which prioritize scalability and environmental safety, have become more popular. A 96.6% yield and > 99% purity were attained by nontoxic solvents such as 1,3-dioxolane–water systems (Wongmoon and Napathorn 2022). Under mild aqueous conditions, biomass breakdown is facilitated by enzymatic hydrolysis employing cellulases and hemicellulases (Jia et al. 2024). Ethyl acetate and butyl acetate are examples of biocompatible solvents that offer good recovery (> 95%) and purity without sacrificing the molecular weight of the polymer (Aramvash et al. 2015). As illustrated in Fig. 3, hybrid approaches that blend solvent efficiency with environmentally friendly methods are becoming increasingly viable options for long-term PHB recovery.

### Green and sustainable recovery approaches

Despite these developments, desalination is still necessary before extraction in halophilic systems, which is energy-intensive and considerably increases production costs. Although osmotic lysis reduces the need for harsh chemicals and simplifies downstream processing, the washing and desalination of saline biomass can require a large amount of freshwater. As a result, extremely saline wastewater is created, and treating and recovering salt can significantly increase energy consumption and operational costs. Future research should concentrate on salt recycling, membrane-based desalination technologies, and water-efficient recovery mechanisms to improve the sustainability of halophilic PHB production. An increase in the economic and environmental sustainability of halophilic PHB manufacturing will require the integration of green solvent or enzymatic technologies and the optimization of salt-tolerant recovery techniques.

### Challenges and future perspectives in halophilic PHB recovery

Despite major breakthroughs in downstream processing, a number of difficulties remain that limit the practical application of halophilic PHB synthesis. Osmotic lysis is regarded as an energy-efficient and environmentally friendly method since it reduces the use of harsh chemicals while facilitating PHB recovery from halophilic microorganisms (Novackova et al. 2022, Kalia et al. 2024). However, washing and desalination of saline biomass may necessitate large volumes of freshwater, resulting in the production of very saline wastewater. The treatment and recovery of salts from these effluents can dramatically increase energy consumption and operational costs, compromising process sustainability. Furthermore, several physical disruption approaches, while effective, have limited scalability and may degrade polymer quality (Balakrishna Pillai et al. 2018, Bhat et al. 2024). Traditional solvent-based extraction technologies also confront issues like toxicity, solvent recyclability, and impact on the environment (Pérez-Rivero et al. 2019). As a result, current research has concentrated on the development of green solvents and environmentally friendly extraction techniques, such as 1,3-dioxolane, ethyl acetate, and other non-halogenated solvents, to improve the effectiveness of recovery while decreasing environmental impact (Abate et al. 2023, Fei et al. 2016, Wongmoon and Napathorn 2022, Aramvash et al. 2015). Future research should focus on salt recycling systems, membrane-based desalination processes, enzymatic recovery methods, and integrated biorefinery approaches to improve the economic and environmental viability of halophilic PHB production (Pérez-Rivero et al. 2019, Jia et al. 2024). Furthermore, pilot-scale techno-economic evaluations and life cycle assessments are necessary to determine the viability of large-scale industrial implementation.

## Recent advancements in PHB research

Metabolic engineering, synthetic biology, omics technology, and computational techniques have propelled recent advancements in PHB research. The two most common approaches are (i) optimizing natural halophilic hosts and (ii) expressing PHB biosynthesis pathways heterologously in industrial microorganisms such as *Escherichia coli*.

Compared with those in parental strains, PHB titers in heterologous systems are increased up to threefold through pathway engineering techniques such as threonine bypass and deregulation of degradation pathways (Lin et al. 2015). According to Table 4, the overexpression of phaCAB or phaEC operons in halophilic hosts increased the PHB content to 70–80 weight% in shake flasks and fed-batch fermentations via food and agro-industrial waste hydrolysates, confirming the viability of genetic enhancement in native strains.

Significant increases in biomass and PHB yields have been made possible by computational techniques that have optimized nitrogen sources, metabolic flux distribution, and carbon allocation (Zhang et al. 2024, Deantas-Jahn et al. 2024, Ramos-Valdovinos et al. 2024). These techniques include flux balance analyses and enzyme-constrained genome-scale models (ecGEMs). By combining artificial neural networks with response surface methods, hybrid machine learning models have improved PHB synthesis while reducing undesirable biomass accumulation by further optimizing the medium composition and environmental conditions (Al Rawahi et al. 2025).

Specifically, programmable changes in halophilic hosts have been made possible by CRISPR/Cas-based genome editing. This has improved PHB and PHBV yields in high-salinity, nonsterile environments by fine-tuning the polymer composition (e.g., increasing the 3-hydroxyvalerate content in *H. bluephagenesis* TD01 by 16-fold). PHB production is also being advanced via circular and sustainable solutions. Environmentally friendly and economical PHB synthesis has been made possible by the co-substrate use of acid whey and non-recyclable fiber waste, as well as the recycling of biodegradable polyester via chemiotechnology techniques (Jia et al. 2024, Wang et al. 2024, Faruga et al. 2025). Furthermore, two-stage bioprocesses and bioelectrochemical systems have used CO₂-fixing bacteria, such as cyanobacteria and purple non-sulfur bacteria, to create PHB, indicating new avenues for the synthesis of carbon-neutral biopolymers (Lee and Salleh 2025).

**Table 4:** Genetic and metabolic engineering strategies to increase PHB production in halophiles

Halophilic bacterial species	Genetic modification	Target gene(s)/pathway	Method	Result	Reference
*Halomonas* spp.	Metabolic pathway manipulation and gene deletion	phaCAB operon, competing carbon sink pathways	Gene deletion, plasmid-based overexpression	Enhanced PHA yield and improved carbon flux	(Paduvari and Somashekara 2025)
*Halomonas* spp. TD01	Repression of competing metabolic pathways	Citrate synthase, TCA cycle–related genes	CRISPRi	Increased PHA accumulation via carbon redirection	(Tao et al. 2017)
Halophilic bacteria	Engineering aromatic compound utilization	Lignin-derived aromatic degradation pathways	Metabolic engineering	Expanded substrate range for PHA synthesis	(Wang et al. 2025)
Halophilic bacteria	Posttranscriptional regulation tuning	mRNA stability of pha genes	Regulatory circuit modification	Improved regulation of PHA biosynthesis	(Peregrina et al. 2021)
Halophilic bacteria	Synthetic biology tool development	Central carbon metabolism & PHA pathways	CRISPR, synthetic circuits	Enhanced robustness and biomanufacturing potential	(Ye et al. 2023)
Halophilic bacteria	Chassis optimization for PHA production	PHA biosynthesis & stress tolerance genes	Genetic and metabolic engineering	Improved suitability as industrial PHA cell factories	(Mitra et al. 2020)
Marine halophilic bacteria	Screening and pathway enhancement	Native pha genes	Isolation and genetic characterization	Identification of efficient bacterial PHA producers	(Sathiyanarayanan et al. 2017)
Halophilic alkane-degrading bacteria	Stress adaptation and energy optimization	Alkane degradation & energy metabolism genes	Pathway-level engineering	Improved tolerance under extreme conditions	(Park and Park 2018)
Halophilic bacteria	Chassis development for bioproduction	Central metabolism and PHA synthesis genes	Synthetic biology approaches	Establishment of robust production platforms	(Zhang et al. 2018)
*Halomonas* spp.	Genome-scale metabolic optimization	Carbon flux and redox balance pathways	CRISPR, plasmid-based tools	Increased PHA productivity and robustness	(Coimbra et al. 2025)
Halophilic bacteria	Regulatory network manipulation	Global regulators of the pha operon	Genetic and regulatory engineering	Improved control over PHA accumulation	(Mitra et al. 2022)
*Halomonas* spp.	Pathway engineering for bioplastics	phaCAB operon, acetyl-CoA supply pathways	Gene overexpression and deletion	Enhanced PHB/PHA synthesis	(Xiao-Ran et al. 2018)
Halophilic bacteria	Regulatory circuit modification	PhaR, PhaP, and global transcriptional regulators	Genetic modification	Increased polymer accumulation efficiency	(Velázquez-Sánchez et al. 2020)
Halophilic bacteria	Copolymer biosynthesis enhancement	PHA synthase variants	Genetic engineering	Production of tailored PHA copolymers	(Delamarre 2004)
Non-model halophilic bacteria	Genome reduction	Nonessential metabolic genes	Genome editing	Increased metabolic efficiency	(Ravagnan and Schmid 2024)
gram-negative halophilic bacteria	Pathway mutagenesis	Central metabolism genes	Transposon mutagenesis	Improved engineering flexibility	(Martínez-García et al. 2014)
*Halomonas* spp.	Genome editing capability establishment	Multiple chromosomal loci	CRISPR/Cas9	Efficient and precise genome editing	(Qin et al. 2018)

Genetic engineering methods, such as CRISPR/Cas systems and metabolic pathway modification, increase carbon flux to PHB biosynthesis and polymer production. Nonetheless, inadequate genetic tractability in several halophilic strains remains a significant hurdle to industrial application. Overall, halophilic microbial platforms have been converted into predictive, stable, and high-yielding systems for industrial PHB production by combining metabolic engineering, computational design, genome editing, and sustainable feedstock usage.

## LCA, PHB production, and industrial potential

According to life cycle assessments (LCAs), PHB has several advantages over traditional plastics (PP, PE, and PET) in environmental terms, as illustrated in Table 5. These advantages include lower greenhouse gas emissions; biodegradability in soil, marine, and home compost environments; and compatibility with circular economy strategies such as recycling and monomer recovery (Harding et al. 2007, Choi and Lee 1999, Koller and Mukherjee 2022). The utilization of waste or renewable feedstocks, such as lignocellulosic biomass, food processing byproducts, and agro-industrial residues, improves sustainability even further by reducing feedstock prices and keeping organic waste out of landfills. Green recovery techniques, such as supercritical CO₂ extraction and enzymatic digestion, can reduce energy use by as much as 40%, making them easier to incorporate into biorefinery operations (Khosravi-Darani et al. 2013).

Compared with traditional non-halophilic producers, such as *Cupriavidus necator* and *Bacillus species*, halophilic bacteria have clear benefits. High-salinity growing conditions facilitate the consistent production of high-quality PHB granules, simplify downstream recovery through osmolysis, and confer inherent resistance to contamination (Mitra et al. 2020, Van-Thuoc et al. 2012). Furthermore, halophiles can enable economically viable and sustainable bioprocessing by metabolizing inexpensive substrates such as molasses, whey, and agricultural leftovers (Obruča et al. 2022).

**Table 5:** Comparative analysis of the physicochemical and processing properties of PHB from halophilic and conventional bacteria

Parameter	Halophilic PHB	Conventional PHB	Remarks	Reference
Molecular weight (Mw)	Medium to high Mw PHB from *Halomonas elongata*,* Halomonas venusta*, and *Salinicola salarius*	High-Mw PHB from non-halophilic producers	Comparable MW suitable for thermoplastic processing	(Abdelrahman et al. 2024, Cristea et al. 2018, Stanley et al. 2020)
Thermal stability (Tm, Td)	Stable up to ~ 170–180 °C	Typical PHB melting range is 175–180 °C.	Thermal behavior is comparable between sources	(Chen and Zhang 2014, Stanley et al. 2020, Obruča et al. 2022)
Crystallinity	Moderate to high crystallinity	High crystallinity (60–80%)	Crystallinity influences brittleness in both PHBs	(Stanley et al. 2020, Cristea et al. 2022)
Mechanical strength	Comparable tensile strength to conventional PHB	High tensile strength but brittle	The host has a limited effect on mechanical strength	(Stanley et al. 2020, Cristea et al. 2022)
Biodegradability	Readily biodegradable in soil and compost	Readily biodegradable	No major difference in biodegradation behavior	(Chathalingath et al. 2023, Rekhi et al. 2022)
Polymer purity	High purity due to osmotic cell lysis and easy recovery	Requires solvent-intensive extraction	Major downstream processing advantage	(Chen and Zhang 2014, Rathi et al. 2013)
Production robustness	Stable under high salinity; contamination-resistant	Sensitive to contamination	Key industrial advantage of halophilic bacteria	(Obruča et al. 2022, Koller and Mukherjee 2022)
Substrate flexibility	Agro-industrial wastes (paddy straw, saline wastes)	Mostly refined substrates	Improves sustainability and cost efficiency	(Abdelrahman et al. 2024, Naitam et al. 2022)
Processing limitations	Salt removal is required before melt processing	No salt-related constraints	A desalting step is needed for halophilic PHB	(Mahansaria et al. 2020, Koller 2022)
Environmental sustainability	Lower sterilization and water demand	Higher energy and sterilization demand	Halophilic PHB better aligns with the circular bioeconomy	(Longo et al. 2024, Rekhi et al. 2022)

Halophilic system drawbacks include low genetic tractability, which might hinder strain optimization, the corrosion of traditional equipment, and the treatment and disposal of salty effluents (Hezayen et al. 2000). Furthermore, saline wastewater management and desalination operations may raise the water footprint and energy requirements of industrial-scale PHB manufacturing. Thus, a complete life cycle assessment (LCA) and techno-economic analysis are necessary to accurately estimate the environmental advantages of halophilic systems. Despite these difficulties, model non-halophiles such as *C. necator* present relatively high maximum yields (~ 85 wt%) and robustness across a variety of substrates, whereas halophiles present competitive PHB yields (50–65 wt%) on inexpensive substrates and facilitate streamlined downstream processing (Getino et al. 2024, Boy et al. 2021).

Overall, halophilic PHB producers offer a viable platform for the synthesis of high-performance, economical, and sustainable biopolymers, especially in applications that require easy processing, environmental compliance, and waste stream valorization. Their distinct features offer industrial flexibility for the widespread use of PHB in packaging, healthcare, and agricultural applications, complementing traditional high-yield methods.

## Regulations and future-oriented applications of PHB

The growing environmental burden of conventional petroleum-derived plastics has fueled global interest in biodegradable alternatives such as polyhydroxybutyrate (PHB). Regulatory support and increasing market demand are important driving forces behind the commercialization of PHB-based products. Biodegradable polymers are being developed and adopted around the world through initiatives such as single-use plastic bans, green public procurement rules, tax incentives, and research funding programs. For example, India’s statewide ban on some single-use plastic products, which took effect in 2022, has provided considerable prospects for PHB-based packaging materials and disposable products. Similarly, the European Union’s Single-Use Plastics Directive and the European Green Deal both promote the use of sustainable bioplastics in packaging, agricultural, and consumer uses. Several governments in North America and Asia have also implemented legislative frameworks and financial incentives to promote bioplastic production and circular bioeconomy initiatives (Koller 2022, Lopez-Arenas et al. 2017).

Compared to many commercially available bioplastics, including polylactic acid (PLA), which frequently requires industrial composting conditions, PHB is more biodegradable in a variety of habitats, including soil, freshwater, marine ecosystems, and composting systems. PHB’s excellent biocompatibility, bioresorbability, and mechanical properties have made it a popular choice for applications in food packaging, agricultural films, controlled-release fertilizers, wound dressings, tissue engineering scaffolds, and 3D-bioprinted medical devices (Chen et al. 2021, Bugnicourt et al. 2014, Rai et al. 2011). In the biomedical arena, PHB’s non-toxic breakdown products make it more suitable for long-term implanted uses.

Recent breakthroughs in material engineering have greatly broadened the functional uses of PHB. Nanomaterials like silver nanoparticles, TiO₂, ZnO, graphene oxide, chitosan, and cellulose-based fillers improve mechanical strength, barrier properties, antimicrobial activity, and sensing capabilities in packaging systems (Chauhan and Chinmay, Bendi 2025). Furthermore, PHB’s intrinsic piezoelectric properties have prompted research into new applications such as flexible electronics, biosensors, wearable devices, and biodegradable energy storage systems (Chen et al. 2021).

Despite these hopeful achievements, significant difficulties remain in the way of PHB’s widespread commercialization. High production costs, restricted thermal processability, brittleness, and a lack of specific industrial composting and waste management infrastructure are still significant impediments. Furthermore, cross-country regulatory harmonization is limited, and standardized certification standards for biodegradability and compostability are not universally implemented. As a result, future research should focus on lowering production costs through waste valorization, enhancing polymer performance through copolymerization and composite engineering, and developing comprehensive regulatory frameworks to assist market penetration.

Sustainability and circularity are widely viewed as critical components of future PHB production systems. Utilizing agro-industrial leftovers, co-substrate fermentation techniques, CO₂-fixing microorganisms, and chemical or biological recycling technologies can significantly reduce environmental impacts and improve process economics (Wang et al. 2024, Khosravi-Darani et al. 2013). The integration of these techniques into circular biorefinery frameworks is expected to improve the long-term viability of PHB production. Overall, sustained technological innovation, supportive laws, and sustainable production techniques will be important in establishing PHB as an economically viable alternative to traditional plastics.

## Conclusion

The growing environmental worries over petroleum-based plastics have highlighted the significance of producing sustainable, biodegradable polymers, with polyhydroxybutyrate (PHB) being one of the most promising options. This review shows that halophilic microorganisms have distinct advantages for PHB production, such as the ability to grow in hypersaline conditions, lower contamination risks during open, non-sterile cultivation, simplified downstream recovery via osmotic lysis, and compatibility with low-cost, waste-derived substrates. Recent breakthroughs in understanding halophilic metabolic pathways, substrate usage, fermentation optimization, reactor design, and downstream processing have resulted in increased PHB production while improving process sustainability.

The use of agro-industrial residues, food-processing wastes, glycerol, lignocellulosic biomass, and other renewable feedstocks in PHB synthesis promotes circular bioeconomy principles by lowering both production costs and waste disposal. Furthermore, advances in metabolic engineering, CRISPR/Cas-based genome editing, systems biology, synthetic biology, and computational metabolic modeling have considerably increased prospects for enhancing carbon flux, polymer yield, and customized biopolymer manufacturing. Green extraction technologies, such as osmotic lysis, enzymatic recovery, and ecologically friendly solvent systems, enhance the environmental sustainability of halophilic PHB production.

Despite these advancements, various obstacles continue to impede large-scale industrial application. High production costs, saline wastewater treatment, equipment corrosion, polymer brittleness, restricted thermal processability, and insufficient pilot-scale techno-economic and life cycle assessments all pose substantial challenges to commercialization. Addressing these constraints would necessitate multidisciplinary initiatives that combine strain engineering, efficient bioprocesses, sustainable downstream recovery, material modification, and integrated biorefinery strategies.

Overall, halophilic PHB synthesis provides a strong and sustainable foundation for next-generation bioplastic production. Continued technological innovation, supportive regulatory frameworks, waste valorization methods, and industrial-scale process optimization will be required to transform laboratory advancements into commercially viable production systems. Such advancements have the potential to drastically reduce reliance on fossil-derived plastics while also helping to achieve global sustainability goals, safeguard the environment, and progress the circular bioeconomy.

## Data Availability

Not applicable.
